# Global Self-Regulation of the Cellular Metabolic Structure

**DOI:** 10.1371/journal.pone.0009484

**Published:** 2010-03-02

**Authors:** Ildefonso M. De la Fuente, Fernando Vadillo, Alberto Luís Pérez-Samartín, Martín-Blas Pérez-Pinilla, Joseba Bidaurrazaga, Antonio Vera-López

**Affiliations:** 1 Department of Mathematics, University of the Basque Country, Vizcaya, Spain; 2 Department of Applied Mathematics and Statistics, University of the Basque Country, Vizcaya, Spain; 3 Department of Neurosciences, University of the Basque Country, Vizcaya, Spain; 4 Department of Health, Basque Government, Vizcaya, Spain; Virginia Tech, United States of America

## Abstract

**Background:**

Different studies have shown that cellular enzymatic activities are able to self-organize spontaneously, forming a metabolic core of reactive processes that remain active under different growth conditions while the rest of the molecular catalytic reactions exhibit structural plasticity. This global cellular metabolic structure appears to be an intrinsic characteristic common to all cellular organisms. Recent work performed with dissipative metabolic networks has shown that the fundamental element for the spontaneous emergence of this global self-organized enzymatic structure could be the number of catalytic elements in the metabolic networks.

**Methodology/Principal Findings:**

In order to investigate the factors that may affect the catalytic dynamics under a global metabolic structure characterized by the presence of metabolic cores we have studied different transitions in catalytic patterns belonging to a dissipative metabolic network. The data were analyzed using non-linear dynamics tools: power spectra, reconstructed attractors, long-term correlations, maximum Lyapunov exponent and Approximate Entropy; and we have found the emergence of self-regulation phenomena during the transitions in the metabolic activities.

**Conclusions/Significance:**

The analysis has also shown that the chaotic numerical series analyzed correspond to the fractional Brownian motion and they exhibit long-term correlations and low Approximate Entropy indicating a high level of predictability and information during the self-regulation of the metabolic transitions. The results illustrate some aspects of the mechanisms behind the emergence of the metabolic self-regulation processes, which may constitute an important property of the global structure of the cellular metabolism.

## Introduction

Living cells are essentially dynamic reactive structures formed by complex membranes surrounding a singular fluid mixture where millions of different biochemical elements interact. In this complex mixture most of the molecules are incessantly synthesized and destroyed through a labyrinthine network of biochemical reactions densely integrated forming one of the most complex dynamical systems in the nature [1], [2].

The enzymes are the most outstanding molecules of this surprising biochemical reactive machinery. They are responsible for almost all the biomolecular transformations, which considered globally are called cellular metabolism.

In the conditions prevailing inside the cell, the enzymes do not work in an isolated way but forming molecular associations, e.g., the analysis of proteome of *Saccharomyces cerevisiae* has shown that 83% of their proteins form complexes containing from two to eighty-three proteins, and its whole enzymatic structure is formed by a modular network of biochemical interactions between protein complexes [3].

Nowadays, there are enough experimental data showing the existence both in prokaryotic and eukaryotic cells of numerous functional enzymatic associations belonging to metabolic pathways like: glycolysis, protein synthesis, lipid synthesis, purine synthesis, Krebs cycle, urea cycle, respiratory chain, fatty acid oxidation, DNA and RNA synthesis, amino acid metabolism, cyclic AMP degradation, etc. [4]–[13].

In addition, reversible interactions of enzymes with structural proteins and membranes are a common occurrence. This results in the existence of microcompartments within the soluble phases of cells. The microcompartmentation provides, on one hand, biophysical and biochemical mechanisms of physiological importance for the regulation of metabolic pathways, and on the other hand, direct transfers of the intermediate substrates from one enzyme to an adjacent enzyme in a process that is called metabolite channelling [14]–[17].

Extensive studies of cellular metabolism during the last three decades have shown that the functional enzymatic associations, the microcompartmentation of the metabolic processes and the metabolite channelling are the principal ways of microstructural organization of cell metabolism [18]–[24].

The cellular organization at the molecular level presents another relevant dynamic characteristic: the emergence of dissipative catalytic patterns.

Experimental observations have shown that the enzymes may form functional catalytic associations in which molecular oscillations may spontaneously emerge. When the oscillations in an enzymatic association are periodic [25]–[29] all the metabolic intermediaries oscillate with the same frequency but different amplitudes [25].

This new type of supramolecular self-organization that operates far from equilibrium conditions was called dissipative structures by Prigogine [29]–[30].

Numerous experimental observations both in prokaryotic and eukaryotic cells have shown the spontaneous emergence of molecular oscillations in most of the fundamental metabolic processes. For instance, there are oscillatory biochemical processes involved in: intracellular free amino acid pools [31], biosynthesis of phospholipids [32], cytokinins [33], cyclins [34], transcription of cyclins [35], gene expression [36]–[39], microtubule polymerization [40], membrane receptor activities [41], membrane potential [42], intracellular pH [43], cyclic AMP concentration [44], ATP [45], respiratory metabolism [46], NAD(P)H concentration [47], glycolysis [48], intracellular calcium concentration [49], metabolism of carbohydrates [50], beta-oxidation of fatty acids [51], metabolism of mRNA [52], tRNA [53], proteolysis [54], urea cycle [55], Krebs cycle [56], mitochondrial metabolic processes [57], nuclear translocation of the transcription factor [58], amino acid transports [59], peroxidase-oxidase reactions [60], photosynthetic reactions [61], and protein kinase activities [62].

To get a more accurate understanding of the global metabolic phenomena, we have developed a reactive dynamical system called dissipative metabolic networks (DMNs) which is basically formed by functional enzymatic associations that may present both steady states and oscillatory molecular patterns.

We define as a metabolic subsystem any group of dissipatively structured functional enzymatic associations that form a catalytic entity as a whole, in which the activity is autonomous with respect to the other enzymatic associations (they carry out their activities relatively independently between them) and molecular oscillations and steady states may emerge spontaneously.

The presence of some regulatory enzymes (both of the allosteric modulation and covalent interaction kind) in each metabolic subsystem makes possible the interconnection among them. Allosteric regulation is the major mechanism by which the enzymatic activities are controlled in cells; they are modulated by means of effectors which are not binding at the active site (catalytic site), but at another locus on the surface of the enzyme (allosteric site) [63]. Such types of reversible modulation may be both positive (activation of their catalytic rates) and negative (inhibition of the reactive process). Covalent modulation allows an active enzymatic form to be converted into an inactive form by covalent modifications and vice versa; this regulation generates “all or nothing” answers [63].

In agreement with all these considerations, a DMN is an open metabolic system formed by a set of discrete modules of functionally associated enzymes (metabolic subsystems) interconnected by substrate fluxes and regulatory signals (allosteric and covalent modulations) in which both steady states and oscillatory catalytic patterns can emerge.

In 1999 we found a singular global metabolic structure able to self-organize spontaneously, characterized by a set of metabolic subsystems always locked into active states (metabolic cores) while the rest of the catalytic elements present dynamics of *on-off* changing states (structural plasticity) [64]. In this theoretical first work with DMNs we also suggested that this cellular metabolic structure could be present in all living cells.

In 2004 and 2005 the existence of this global metabolic structure was verified for *Escherichia coli*, *Helicobacter pylori*, and *Saccharomyces cerevisiae* under different growth conditions by means of flux balance analysis applied to metabolic networks and it was also suggested that this self-organized enzymatic configuration appears to be an intrinsic characteristic of metabolism, common to all living cellular organisms [65]–[67].

We have investigated the influence of some molecular processes in the self-organization of the global metabolic configuration by means of numeric simulations in DMNs and we have observed an asymptotic trend approximately 100% of the networks displaying this kind of global configuration when the number of metabolic subsystems is incremented: this suggested that the number of catalytic elements could be the fundamental element for the emergence of this global metabolic behaviour [68].

Recently, we have performed extensive DMNs simulations (around 15,210,000 networks) taking into account: the proportion of the allosterically regulated enzymes and covalent enzymes present in the networks, the variation in the number of substrate fluxes and regulatory signals per catalytic element, the random changes in the topology of all flux and regulatory signal interconnections, as well as the random changes in the values of the outer fluxes. The results show that the fundamental factor for the spontaneous emergence of this global self-organized enzymatic structure is the number of catalytic elements in the metabolic networks [69].

Here, our main goal is to get a more accurate understanding on the dynamical features which guarantee that the global functional structure is preserved under different external inputs. For this aim, we have studied by means of non-linear dynamic tools, different transitions in the catalytic patterns belonging to a dissipative metabolic network. Concretely, we have analyzed by means of non-equilibrium statistical physics tools the following: the stability of the catalytic activities by means of the Lyapunov exponent, the type of signal belonging to the complex transitions by calculating the slope of the power spectral, the rate of entropy production by testing the Approximate Entropy and the possible presence of long-term correlations in the enzymatic transitions data by means of the bridge detrended scaled windowed variance analysis.

Here we have also studied both simple and complex dynamical transitions of different metabolic subsystems and we have reconstructed the complex attractors responsible for the dynamical regulations during the transitions in the metabolic activities.

Our analysis has shown the spontaneous emergence of self-regulation processes in the metabolic patterns. Likewise, we have observed that the complex numerical series analyzed correspond to fractional Brownian motion. They exhibit long-term correlations and low Approximate Entropy indicating a high level of predictability and information during the self-regulations of the metabolic transitions.

These results illustrate some aspects in the mechanisms behind the emergence of the metabolic self-regulation processes which may constitute an important property of the global structure of the cellular metabolism.

## Methods

### 1. Dissipative Metabolic Networks Model

Dissipative metabolic networks (DMNs) are dynamical systems basically formed by a given number of interconnected metabolic subsystems. Each metabolic subsystem represents a group of dissipatively structured functional enzymatic associations (the catalytic processes can present both stationary and oscillatory activity patterns and comprise an infinite number of distinct activity regimes). These enzymatic sets are considered as individual catalytic entities and receive both input fluxes (the substrates of the enzymatic reactions) and regulatory signals, which may be of three types: activatory (positive allosteric modulation), inhibitory (negative allosteric modulation) and all-or-nothing type (which correspond with the regulatory enzymes of covalent modulation).

Input-output conversion is made in two stages. First, the input fluxes are transformed into an intermediary activity by means of flux integration functions. In a second phase, the “intermediary activity” is modified by means of the “regulatory signals integration”, which depends on the combination of the received regulatory signals. Each regulatory signal has an associated regulatory coefficient which defines the intensity of its influence.

In DMNs, when the set of dissipatively structured enzymes shows an activity with a rhythmic behaviour the output activities present nonlinear oscillations with different levels of complexity as could be expected in the cellular conditions “in vivo”.

Formally, we assume that the activity of the i-th metabolic subsystem is defined by 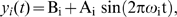
where 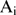 is the amplitude of oscillation, 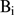 is the baseline and 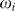 is the oscillation frequency. Moreover, in order that 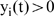 we assumed that 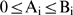 and we also suppose that the means and the frequencies are bounded values, so there exist 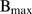 and 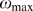 such that
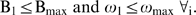
In this way, the activity of each metabolic subsystem 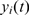 can be characterized by three variables 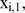 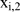 and 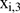, with values between 0 and 1 such that
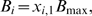


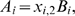


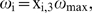
The subsystem is inactive when 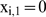, and is steady state when 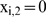 or 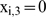.

Fix 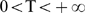 and let 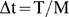 be a time interval during which the oscillations are constants, in the m-th time interval between 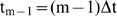 and 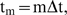 the activity of the i-th subsystem is represented by the vector 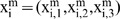 and the state matrix
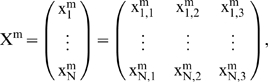
characterizes the whole DMN system, *N* is the total number of subsystems.

To study the evolution of the whole system, we assume that each subsystem receives two different kinds of inputs:

- The substrates of the biochemical reactions.
- Regulatory signals of three types: activatory, inhibitory and total inhibitory.

These inputs may produce a change in the activity of the subsystems. Moreover, according to experimental observations, the output activity must be stationary or periodic.

Each subsystem processes inputs to produce outputs in two stages:

1. An intermediate activity is obtained using the flux integration functions.
2. The received regulatory signals originate a regulatory signal integration which varies the intermediary activity.

### 2. Flux Integration

Let us suppose that the i-th subsystem receive flux from the j-th, its intermediate values 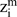 will be computed by three flux integration functions
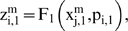


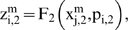


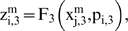
Where 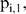 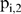 and 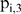 are parameters associated to the flux integration function which are characteristic of each metabolic subsystem, and the 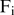 are piecewise linear approximations for nonlinear functions obtained in [70] by Goldbeter and Lefever in their studies about the oscillations for glycolytic subsystems. In this paper, the functions will be the following:
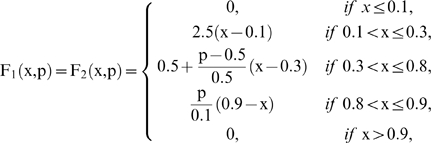
and
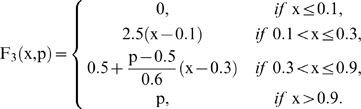
When a subsystem receives flux from at least two subsystems, we compute the arithmetic mean.

### 3. Regulatory Signal Integration

In this second stage, the intermediary values are modified using the signal integration functions, which depend on the combination of the received regulatory signals and their corresponding parameters (regulatory coefficients). In the metabolic subsystems, the existence of some regulatory enzymes (both allosteric and covalent modulation) permits the interconnection among them. The allosteric enzymes present different sensitivities to the effectors, which can generate diverse changes on the kinetic parameters and in their molecular structure; likewise, the enzymatic activity of covalent modulation also presents different levels of regulation depending on the sensitivity to other activators or inhibitors.

These effects on the catalytic activities are represented in the dynamical system by the regulatory coefficients and consequently each signal has an associated coefficient which defines the intensity of its influence. There exist three kinds of signal integration functions:

- Activation function AC.
- Inhibition function IN.
- Total inhibition function TI.

In this way, to compute 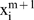 from 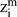 the i-th subsystem receives enzymatic regulatory signals from r subsystems and they work sequentially computing
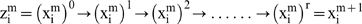
where each step depends on the signal type. From 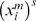 to 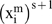 if the signal is AC and is received from the j-th MSb
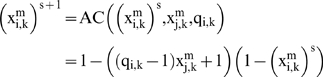
for *k* = 1, 2, 3 and 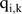 are regulatory coefficient to each allosteric activity signal which represents the sensitivity to the allosteric effectors.

If the allosteric signal is inhibitory
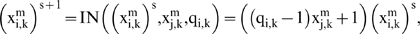
and, finally, if the signal is of the total inhibition type
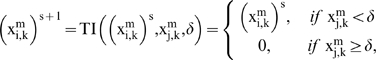
where δ, the threshold value, is the regulatory coefficient associated to each enzymatic activity signal of covalent modulation which defines the intensity of its influence.

### 4. Random Metabolic Network Generation

**Figure 1:**
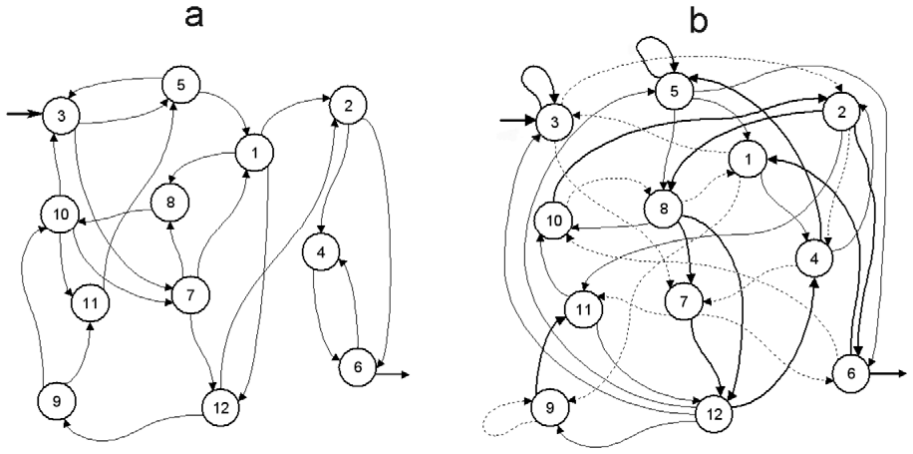
Topology of the metabolic network. Dissipative metabolic network formed by 12 subsystems in which the interconnection by substrate fluxes (figure 1a) and input regulatory signals (figure 1b) are reflected. These regulatory signals, allosteric activation (thin line), allosteric inhibition (thick line) and covalent modulation of total inhibition (dashed line), come from any element of the network and do not require any flux relationship.

First, we have fixed the following elements with control parameters: 12 subsystems in the DMN, 2 substrate input fluxes for each subsystem (figure 1a), 3 input regulatory signals for each metabolic subsystem and the same proportion of the allosteric activation signals, allosteric inhibition signals and regulatory signals of covalent modulation present in the network (figure 1b). DMNs are open systems and certain metabolic subsystems may receive a substrate flux from the exterior. Here, we have arbitrarily fixed the metabolic subsystem number three for this function.

Having fixed these elements, the structure of the network has been randomly configured (following the uniform distribution) including: the topology of flux interconnections and regulatory signals, the 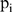 parameters associated to the flux integration functions, the 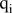 regulatory coefficients to the allosteric activities, and the values of the initial conditions in the activities of all metabolic subsystems (Table S1).

The values of 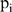 and 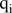 are a random number between 0 and 1. The changes in the parameters 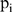 modify the flux integration functions, which are piecewise linear approximations for the nonlinear functions obtained in [70] by Goldbeter and Lefever. The values of 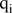 close to 0 represent a low level of influence of the allosteric regulatory signals, and the values of 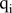 close to 1 represent a high level of influence of the allosteric regulatory signals. The random value of these 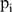 and 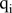 parameters originates metabolic networks with a great variety of activities in each subsystem.

We have taken the constants 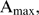 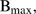 and 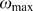 equal 2.

Finally, given *T* and *M* we calculate the activity matrices 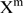 for *m* = 1, …, *M* using the flux integration functions and the regulatory signals.

### 5. Representation of the Activity of the Metabolic Subsystems

The output of our automaton provides a sequence of three positive values 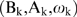 for 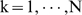 which represent baselines, amplitudes and frequencies respectively. Then we define the activity 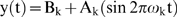 for the time *t* between 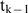 and 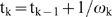. These activities are essentially piecewise linear functions, the baselines plus sinusoids with different amplitudes 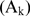 and frequencies 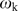 and therefore may be discontinuous at times 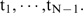


To avoid these discontinuities, per each 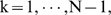 we determine a unique three-degree polynomial *p*(*t*) whose graph passes through the points 
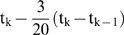
 and 
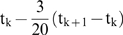
 being its first derivatives in these points equal zero, finally *p*(*t*) plus sinusoids is the activity in a bit interval 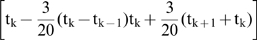.

### 6. Maximal Lyapunov Exponent

To calculate the maximal Lyapunov exponent we have used the Wolf algorithm [71]; its idea is simple: Consider a reconstructed attractor and define an arbitrary starting point 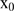 lying on it, one should find another point 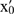, which is close in space but is distant in time to 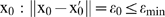 and 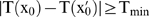. Then trace systems dynamics using initial points 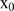 and 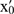. Then a distance 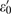 between two trajectories will exceed some value 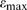, stop and fix time of tracing 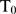 and ratio 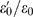. After that one should find another starting point 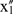, which is close to 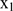 and shifted in the direction of the vector 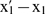. Let 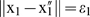. Trace the dynamics of the system using initial points 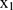 and 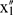. Then a distance 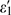 between two trajectories will exceed 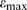, stop and fix time of tracing 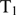 and ratio 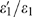, etc.

The Maximal Lyapunov exponent is estimated as 
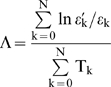
where N is the iteration number.

### 7. Scaled Windowed Variance Analysis (bdSWV)

This method generates an estimation of the Hurst exponent (H) for each series.

According to this procedure, if the signal is of the form 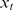, where t = 1,…,*N*, then the following steps are carried out for each one of the window sizes *n* = 2,4,…,*N*/2,*N* (if *N* is not a power of 2, then *n* takes the values 2,4,…,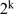, where *k* is the integer part of 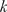 *N*):

1. Partition of the data points in 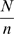 adjacent non-overlapping windows 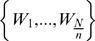 of size *n*, where 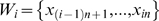. If *N* is not a power of 2 and *N* is not divisible by *n*, then the last remaining points are ignored for this value of *n*. For instance, if *N* = 31 and *n* = 4, the first 28 points are partitioned into seven windows.
2. Subtraction of the line between the first and last points for the points in the n-th window.
3. For each 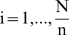, calculation of the standard deviation 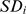 of the points in each window, by using the formula
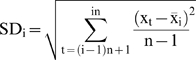
(1)where 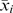 is the average in the window 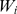.
4. Evaluation of the average 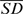 of the 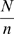 standard deviations corresponding to equation [1].
5. Observation of the range of the window sizes *n* over which the regression line of 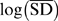 versus 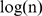 gives a good fit (usually some initial and end pairs are excluded).
6. In this range, the slope of the regression line gives the estimation of the Hurst coefficient *H*.

The empirical range of windows corresponding to step 5) which we have found to be in accordance with the guidelines appearing in [72], and consequently we have excluded the first two and last three points. We have used in our work the program bdSWV, available on the web of the Fractal Analysis Programs of the National Simulation [73].

## Results

To investigate the elements that may affect the catalytic dynamics under a global metabolic structure characterised by present metabolic cores, we have performed a unique DMN, which is later perturbed, to study its reactive dynamical answer.

In this metabolic network, first we have fixed the following parameters:

- The number of subsystems in the DMN: 12.
- The number of substrate input fluxes for each subsystem: 2 (figure 1a).
- The number of input regulatory signals for each metabolic subsystem: 3 (figure 1b); these regulatory signals (allosteric activation, allosteric inhibition and covalent modulation of total inhibition) come from any element of the network and do not require any flux relationship. Therefore, there are not metabolic subsystems with single fluxes and every metabolic subsystem receives fluxes and regulatory signals.
- The same proportion of the allosteric activation signals, allosteric inhibition signals and regulatory signals of covalent modulation present in the network.

DMNs are open systems, and certain metabolic subsystems may receive a substrate flux from the exterior. Here, we have arbitrarily fixed the metabolic subsystem number three for this function.

After determining these characteristics of the network, its architecture was randomly configured including: the topology of flux interconnections and regulatory signals, the parameters associated to the flux integration functions and regulatory coefficients of the allosteric activities, as well as the values of the initial conditions in the activities of all metabolic subsystems (see “Methods” section for more details).

**Figure 2:**
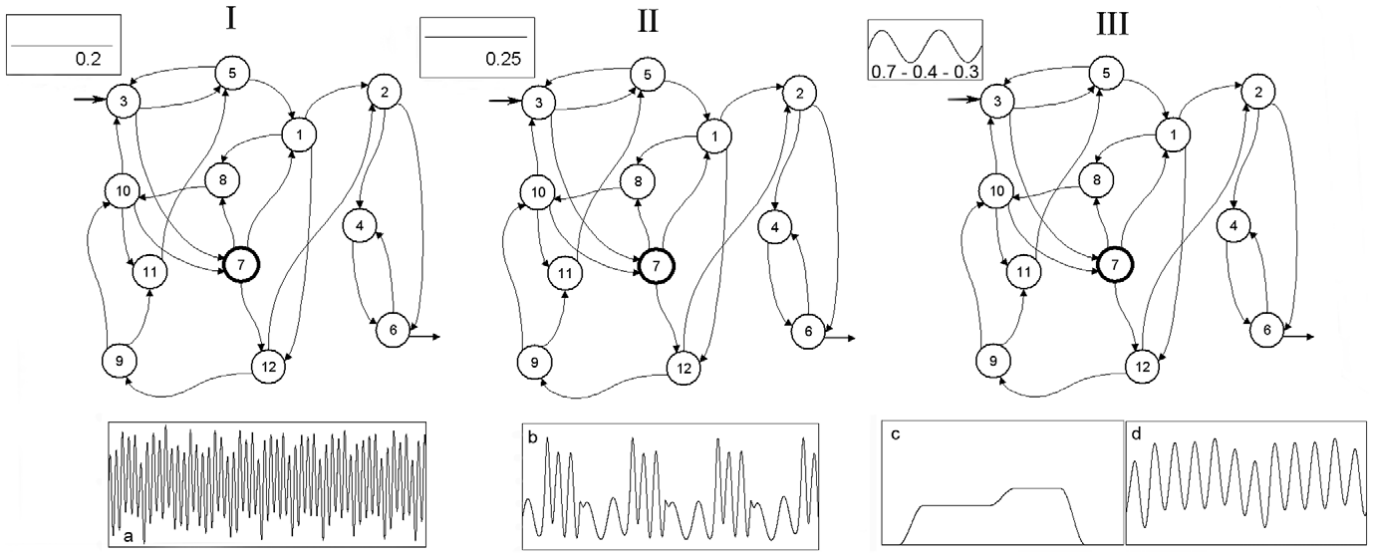
Summary of the numerical experiments. The DMN was perturbed by three different values of the external input flux belonging to the metabolic subsystem number three. I: Chaotic patterns emerge under a stationary external input flux of substrate with a baseline of 0.2 (figure 2a). II: Under a stationary input flux with a baseline of 0.25, the network undergoes a reorganization of its dynamics and spontaneously all the subsystems present complex regular behaviours in their activities (figure 2b). III: Finally, we have considered an oscillatory external input flux (baseline: 0.7, amplitude: 0.4 and frequency: 0.3) and in the metabolic subsystems steady-state patterns (figure 3c) and simple regular transitions between different periodic oscillations (figure 3d) emerge. Circles with thin line represent subsystems always locked into active states, while the circle with thick line represents a metabolic subsystem with dynamics on-off.

Once defined (Table S1), the DMN was perturbed to three different values of the external input flux belonging to the metabolic subsystem number three (figure 2).

We have first studied the dynamical patterns that emerge under a stationary external input flux of substrate with a baseline of about 0.2.

In this case, after the numerical computation, we have observed that all metabolic subsystems present deterministic chaotic activities, i.e., the output activity of all subsystems exhibits infinite transitions, modifying uninterruptedly their activity so that they never repeat themselves for arbitrarily long time periods.

**Figure 3:**
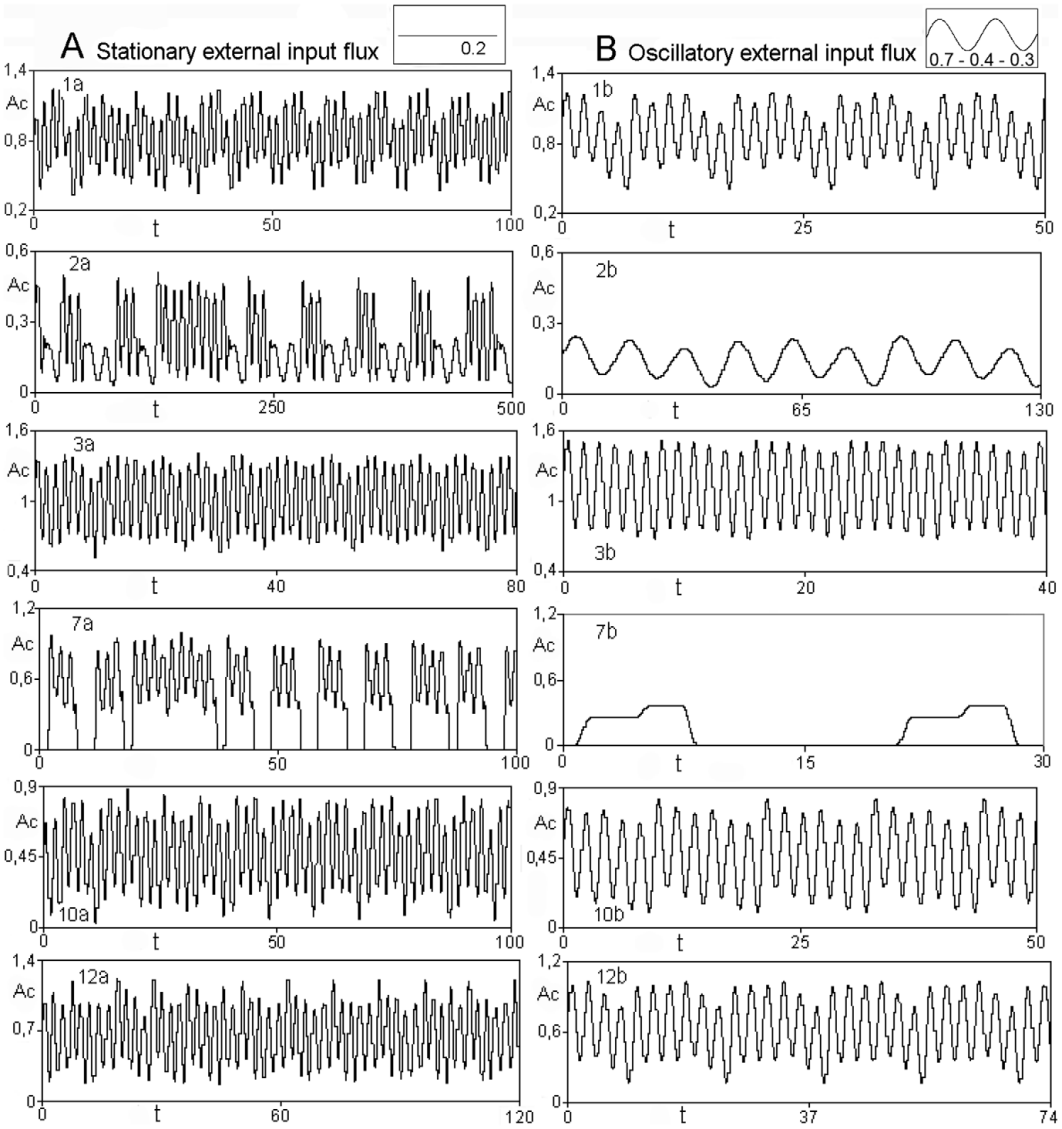
Emergent dynamic behaviours in the dissipative metabolic network. A: chaotic series, the metabolic subsystems show infinite transitions between different activity patterns when the network is perturbed by a stationary external input flux of substrate (baseline of 0.2). B: regular transitions between periodic or steady state behaviours emerge when the network is perturbed by an oscillatory external input flux of substrate (baseline: 0.7, amplitude: 0.4 and frequency: 0.3). The activity Ac developed by each metabolic subsystem is represented as a function of the time t.

In figure 3A are represented the dynamical behaviours of six of them. We can observe that the amplitudes and periods of the oscillations vary considerably from one subsystem to another; for instance, the subset of enzymes number two shows low activity values during irregular oscillations.

More interesting is the behaviour of the metabolic subsystem number seven which eventually exhibits activity zero during irregular intervals of time. This is due to the fact that in the net spontaneously emerges a global functional structure in which 11 subsets of enzymes are always in an active state (metabolic core) whereas the subsystem number seven always exhibits a chaotic dynamic on-off (see [68], [69] for more details for this kind of global structure).

The mechanism that determines these chaotic behaviours is not prefixed in any part of the metabolic system. There is neither feedback with oscillatory properties nor other rules that determine the system to present complex transitions in the output activities of the metabolic subsystems. The complex dynamic behaviours which spontaneously emerge in the network have their origin in the regulatory structure of the feedback loops, and in the nonlinearity of the constitutive equations of the system.

Next, we have considered a stationary input flux of external substrate with a baseline of 0.25 (figure 2).

Under these new conditions, the same net undergoes a reorganization of its dynamics and spontaneously all the subsystems present complex regular behaviours in their activities with large number of transitions between periodic oscillations without steady-states, e.g., the metabolic subsystem 1 exhibits complex activity patterns with 36 maxima and 36 minima per oscillation.

The global functional structure of the network has not been altered by the perturbation; the metabolic core preserves the same number of subsystems always in an active state whereas the subsystem number seven exhibits a dynamics of on-off changing states.

Finally, in the network we have considered an oscillatory external input of substrate in the subsystem number three (baseline: 0.7, amplitude: 0.4 and frequency: 0.3).

Again, the network spontaneously auto-organizes its activity patterns provoking the emergence of a qualitative change in the whole system and the activities of all subsystems presents simple regular transitions between different periodic oscillations or steady-state patterns (figure 3B). For instance, the enzymatic subset number 1 (figure 3B-1b) shows an output activity characterized by presenting uninterrupted transitions between regular waves with six oscillations per period.

The ranges of amplitudes and periods of these regular patterns vary considerably from a subsystem to another. For instance, subsystem number two, under the new conditions shows very low activity with low frequency values.

The subsystem number 7 (figure 3B-7b) exhibits transitions between steady states and its activity is eventually zero during regular intervals of time. This is due to the fact that in the net, the same global functional structure which has not been altered by the perturbation emerges spontaneously.

### Local Attractors in the DMN

The dynamical reorganizations of the metabolic network during the changes provoked by the three different external fluxes (figure 2) originate the emergence of different transition activities which ends in one of the attractor states. For any metabolic subsystem, each attractor represents the set of all the possible asymptotic behaviours.

Formally, if y(t) is the activity of any subsystem in the network, a set A is called an attractor for this subsystem in the following three conditions:

1. It is impossible to go out, in other words, if 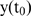 is in A for some time 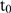, later y(t) remains in A.
2. There exists a neighbourhood of itself B (basin of attraction) such that for any initial condition in B, the system approaches indefinitely A.
3. A is a compact set; this means it is a closed and bounded set.

Consequently, the attractor characterizes the asymptotic catalytic behaviour of each subsystem.

To investigate the dynamics of DMN, we need to reconstruct the attractor from the time series and compute its maximal Lyapunov exponent which is very useful in testing the existence of chaos.

Positive Lyapunov exponent indicates sensitivity to initial conditions, a hallmark of chaos [74], by contrast, the leading Lyapunov exponent would be zero for quasiperiodic evolution or when the system is in some sort of steady state mode. A negative Lyapunov exponent is characteristic of a stable fixed point or stable periodic orbit in the phase space.

To reconstruct attractors we have used time-delay embedding [75]; this technique allows us to establish a phase space representation for time series as a function of the current and of the previous values; for that it requires a delay and an embedding dimension.

Given a time series x(t), t = 1,2,…N, the m-dimensional return map is obtained by plotting the vector 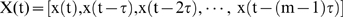 where 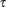 is an integer delay.

**Figure 4:**
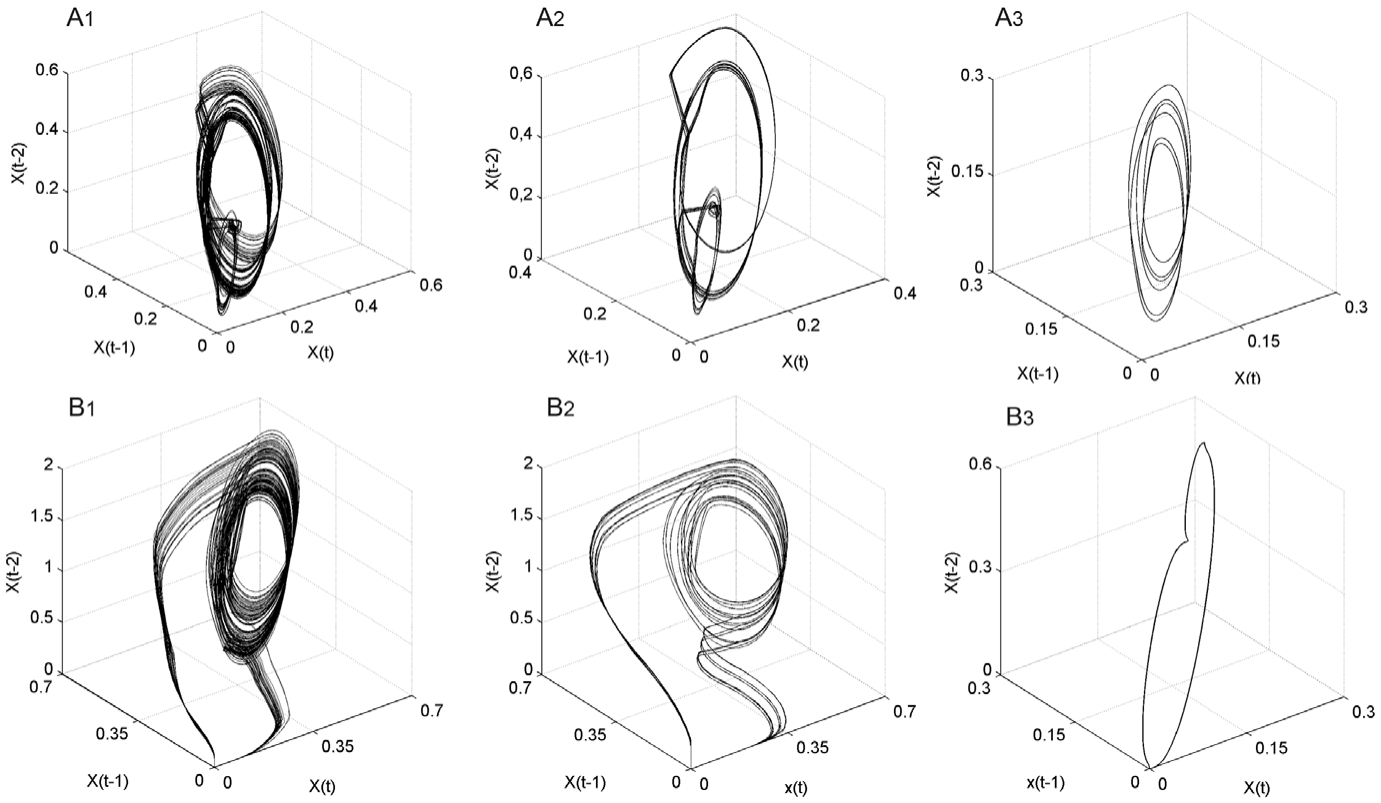
Local attractors in the dissipative metabolic network. The dynamical reorganizations of the network are represented by local attractors which are the set of possible asymptotic behaviours for each metabolic subsystem activity. The first row (A1, A2 and A3) corresponds to subsystem number two during the changes provoked by three different patterns in the external input fluxes, and the second row (B1, B2 and B3) corresponds to subsystem number seven.

This converts the one dimensional vector x(t), into the m-dimensional vector X(t). The dimension m is known as the embedding dimension, and the trajectory of X(t) converges to an attractor in the m-dimensional Euclidian space, which is, up to a continuous change of variable, the attractor of the subunit dynamical system. In figure 4 we can observe six different attractors, the first row corresponding to subsystem number two representing different re-organizations of the activity patterns during the three perturbations, and the second row is the result for the re-organizations of the catalytic activities belonging to subsystem seven.

Once the attractor for each metabolic subsystem has been created, we have computed the maximal Lyapunov exponent with the results of the third column in table 1. The arithmetic mean is about 0.223, the standard deviation 0.07 and the range between 0.122 and 0.319. These results confirm the chaotic behaviour for all subsystems under a stationary external input flux of substrate with a baseline of about 0.2.

**Table 1:** Analysis of the regular and chaotic series for the dissipative metabolic network with 12 metabolic subsystems.

		Chaotic series			Regular	series
MSb	β	H	λ	ApEn	MSb	ApEn
1a	1.663	0.196 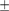 0.090	0.249	0.413	1b	0.044
2a	1.744	0.173 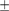 0.077	0.316	0.356	2b	0.012
3a	1.634	0.185 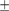 0.096	0.194	0.313	3b	0.052
4a	1.320	0.190 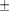 0.096	0.207	0.407	4b	0.035
5a	1.831	0.169 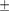 0.095	0.116	0.326	5b	0.024
6a	1.845	0.161 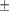 0.092	0.144	0.380	6b	0.040
7a	1.479	0.270 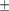 0.089	0.289	0.266	7b	0.028
8a	2.108	0.173 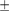 0.092	0.251	0.322	8b	0.045
9a	1.594	0.175 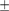 0.094	0.122	0.318	9b	0.036
10a	1.233	0.183 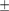 0.091	0.249	0.374	10b	0.042
11a	1.982	0.188 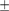 0.086	0.319	0.379	11b	0.041
12a	1.720	0.167 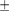 0.092	0.217	0.379	12b	0.047

Msb: the number of metabolic subsystem; β: slope of the power spectral density plot; H: Value of the Hurst exponent; λ: maximal Lyapunov exponent; ApEn: approximate entropy value.

### Long-Term Correlations in the Metabolic Subsystem Activities

In order to study the presence of long-term correlations in the numerical chaotic data, we have determined whether the series is a fractional Gaussian noise (fGn) or a fractional Brownian motion (fBm); fGn and fBm can be distinguished by calculating the slope of the power spectral density plot.

The signal is said to exhibit power law scaling if the relationship between its Fourier spectrum and the frequency is approximated asymptotically by 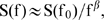 for adequate constants 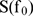 and β. If 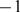
<β<1, then the signal corresponds to an fGn. If 1<β<3, then the signal corresponds to a fBm [76].

**Figure 5:**
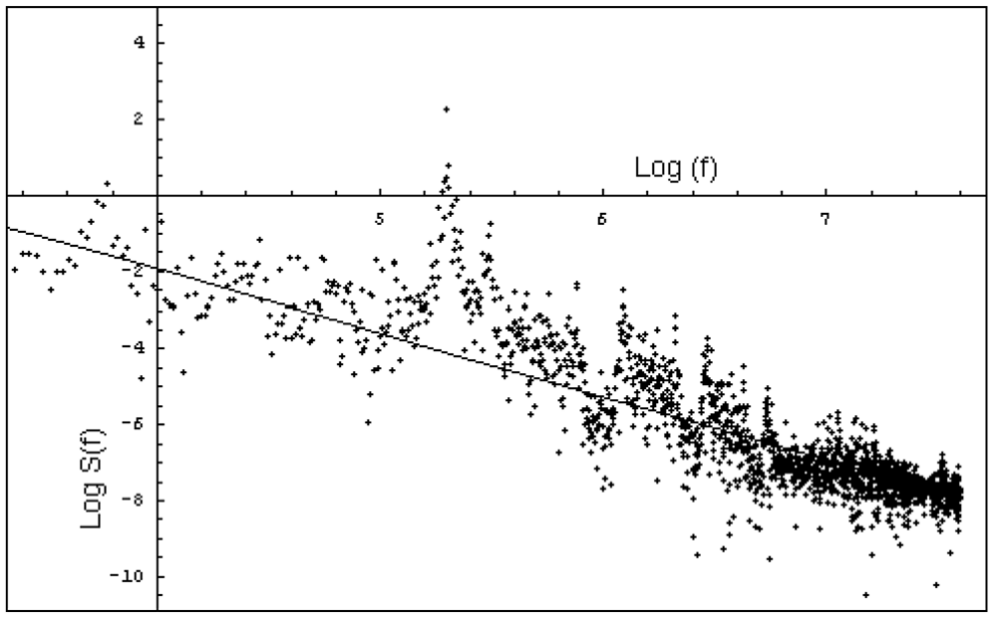
Spectral density plot of a representative series of the dissipative metabolic network. S(f) is the absolute value of the Fourier transform and f the frequency. The opposite of the slope has a value of 1.663; this means that the process is a fractional Brownian motion (fBm).

The regression line was estimated for the pairs (log S(*f*), log *f*), where *f* is the frequency and S(*f*) the absolute value of the Fourier transform (figure 5). The β constant was taken to be the opposite of the coefficient of *x* in that regression line.

The analysis in table 1 show that all the metabolic activity series that we have studied present power law scaling with β in the range 1.233–2.108, which suggests that the series are fBm.

A number of tools are available for estimating the long-term correlations of an fBm time series. The scaled windowed variance analysis is one of the most reliable methods that have been thoroughly tested on fBm signals [71]. In particular, we have used the bridge detrended scaled windowed variance analysis (bdSWV) for the analysis of these temporal sequences of metabolic activities [77].

This method generates an estimation of the Hurst exponent (H) for each series. For a random process with independent increments, the expected value of H is 0.5. When H differs from 0.5, it indicates the existence of long-term memory, that is to say, dependence among the values of the process. If H>0.5, it was produced by a biased random process which exhibits persistent behaviour. In this case, for several previous transitions, an increment on the phase-shift average value implies an increasing trend in the future. Conversely, a previously decreasing trend for a sequence of transitions usually implies a decrease for a similar sequence. Antipersistent behaviour is obtained for 0<H<0.5, a previously decreasing trend implies a probable increasing trend in the future and an increase is usually followed by decreases [71], [77].

We have used in our work the program bdSWV, available on the web of the Fractal Analysis Programs of the National Simulation Resource [72] (see the Methods section for more details).

In table 1 the results of the bdSWV analysis of each one of the 12 metabolic series are shown. All the values of the estimation of the Hurst coefficient H were smaller than 0.5, in all the numerical series. The arithmetic mean of the whole set of series obtained is 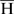 = 0.1858, with a standard deviation 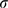 of 0.028 and a range of 0.161–0.196, indicating that the studied metabolic activity exhibits long-term persistence (more concretely, antipersistent behaviour). Therefore, the value of each metabolic activity studied depends to some extent on previous ones.

### Global Attractors in the DMNs

The set formed by the asymptotical behaviours followed by all the metabolic subsystem activities form the global attractor of the metabolic net.

Formally, if 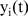 is the activity of any subsystem in the network M, obviously 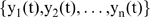 is a dynamical system in 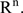


After some transitory time, the activity subsystem functions 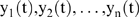 end up and remain in one global attractor representing the catalytic asymptotic behaviour of the whole network. Since these attractors have many dimensions, we have represented their projections in only three dimensions.

**Figure 6:**
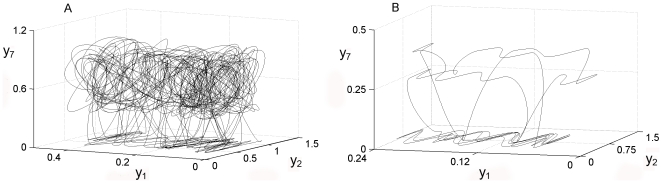
Global attractors in the dissipative metabolic network. 4A: chaotic global attractor which emerge when the network is perturbed by a stationary external input flux of substrate with a baseline of about 0.2. Figure 4B shows a regular global attractor, i.e. the set of biochemical states when the network is perturbed by an oscillatory external input of substrate. We have represented their projections in only three dimensions for the metabolic subsystems 1, 3 and 4 (the x-axis is the 1a series, the y-axis is the 3a series, and the z-axis is the 4a series).

In figure 6 we can see global attractors both chaotic (figure 6a) and metabolic regular dynamics (figure 6b) for subsystems 1, 2 and 7.

The entropy theory of dynamical systems can be found in many textbooks [78]–[79]. Roughly speaking, the entropy will be highest when all transition states have the same number of possible emergency, and the maximum entropy occurs when any transition pattern could be found with equal probability, therefore the entropy will be lowest and information highest when one pattern or a few patterns are dominant (small number of states with high probabilities).

The entropy is also a useful concept in the study of the attractors which may allow estimating the degree of complexity and information contained in them. Concretely, Kolmogorov–Sinai entropy (K–S entropy) provides a measure of the information and the level of predictability in the attractor [80]. However, the K–S entropy cannot be computed directly, it can only be approximated. Problems arise when entropy rates have to be estimated from a finite number of observations containing a relatively high noise component.

A practical solution to this problem has been put forward using a developed family of statistics named Approximate Entropy (ApEn) which is a good approximation of the Kolmogorov-Sinai entropy [81].

Formally, given N data points from a time series x(1), x(2),., x(N), two input parameters m and r must be fixed to compute ApEn, denoted precisely by ApEn(m, r, N).

To estimate ApEn, first we form the m dimensional vector sequences X(1)….X(N-m+1) such that X(i) = (x(i)…..x(i-m+1)) which represent m consecutive values. Let define the distance between X(i) and X(j) (d[X(i),X(j)]) as the maximum absolute difference between their respective scalar components and for each X(i) we count the number of j such that d[X(i),X(j)]<r, denoted as 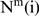 and 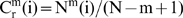, which measure within a tolerance r the frequency of patterns similar to a given one of window length m.

The average value of 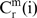, is 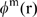 which portrays the average frequency of the ocurrence that all the m-point patterns in the sequence remain close to each other, and finally 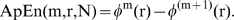
The idea is that ApEn measures the logarithmic likelihood that runs of patterns that are close (within r) for m contiguous observations remain close on subsequent incremental comparisons.

In table 1 the results of the ApEn estimation for the 12 chaotic series are shown. The arithmetic mean is 0.353, with a standard deviation 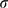 of 0.044 and a range of 0.313–0.413 which indicates high information contained in the global attractor that emerges in the phase space when the studied metabolic network is perturbed by a stationary external input flux of substrate with a baseline of 0.2.

Finally, we have computed the ApEn entropy of the regular series (see table 1) and all the values are near to zero. These values correspond to the expected ones.

In any process, if the series are regular, the KS entropy is very near to zero, when they are chaotic, it grows and it is infinite in numeric series with random noise (82).

## Discussion

In an attempt to investigate the dynamical features of the dissipative metabolic networks which guarantee that the global functional structure is preserved under different external inputs we have studied, using non-equilibrium statistical physics tools, catalytic activities belonging to a dissipative metabolic network.

The first results show that the chaotic numerical series present power-law scaling with a slope of the power spectral density plot β varying between 1.126 and 2.658, which corresponds in all case to fractional Brownian motion (fBm). This fBm is a generalization of Brownian motion in which the increments are normally distributed but they are no longer independent and therefore the process is correlated in time. This dynamic characteristic is also present in different physiological signals [83], [84].

In order to estimate the level of the long-term correlations, the chaotic numerical data were analyzed by means of the bdSWV method finding values for the Hurst exponent (H) in a range of 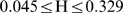.

We clearly found that the values of the Hurst exponent were lower than 0.5 in all cases and consequently, the metabolic processes analyzed depend on their history; they are conditioned to some extent on the previous ones.

Values of H<0.5 are interpreted as characteristic of ‘trend-reversing’ or long-term antipersistence, i.e., the behaviour of the metabolic series tends to reverse itself, an increasing tendency in the past for a sequence of metabolic transitions is followed on average by decreases in the future, and conversely, a decreasing trend in the past is likely to be followed by an increasing tendency.

The chaotic metabolic transitions transmit information and this information presents long-term memory properties. A complex dynamic phenomenon may emerge in the metabolic system during large sequences so that the state of the catalytic activity in each subsystem is affected by a certain number of its previous activity states.

In deterministic chaotic processes, the sensitive dependence on initial conditions will lead to large changes in posterior system states with exponential divergence in the orbits of the chaotic attractors. However, these perturbations do not prevent the integral trajectories belong to the attractor. In fact, the chaotic dynamical system has enough information to allow the trajectories to remain quite close to the attractor. This information can be estimated by means of the Kolmogorov-Sinai entropy which indicates the degree of complexity and information contained in the attractors and estimates the rate at which information about the state of a system is lost. Likewise, this information is correlated in time i.e., it exhibits long-term persistence.

Both properties, sensitive dependence on initial conditions and persistence, coexist in the chaotic dynamic systems. These deterministic systems contain a certain level of information (level of predictability) and this information presents long-term correlation.

Long-term correlations have also been observed in experimental studies, e.g., the quantification of DNA patchiness [85], physiological time series [84], [86], NADPH series [87], DNA sequences [88]–[90], K+ channel activity [91], mitochondrial processes [57] and neural electrical activity [92].

In previous works, we have also studied, by means of the *R/S* method, the relationship between chaotic deterministic processes and long-term correlations. For instance, we have investigated persistent properties in the Lorenz attractor [93], glycolytic attractors (93), chaotic cardiac oscillations [94] and electrical neuronal processes [95]. Likewise, we have also observed long-term antipersistence (*R/S* method) in numerical series of oscillatory amplitudes belonging to a DMN [64].

On another hand, the DMN was perturbed by three different values of the external input flux and as consequence local and global attractors appear spontaneously in the phase spaces belonging to metabolic subsystems and global network.

When a metabolic subsystem begins its activity the catalytic patterns fall into one of the possible local attractors and all the dynamical reactive behaviours become trapped inside. Local attractors determine the output activity of the metabolic subsystems. Changes in their parameters (see Methods section) provoke changes in the type of emergent patterns (either causing a stationary state or an oscillatory behaviour) and in the values of the dynamical activity.

Global attractors determine that the metabolic system operates like an individual and complete integral system, and their dynamics are responsible, on one hand, for the self-organization of the metabolic processes (the emergence of a global functional structure with several sets of enzymes always in an active state, whereas the rest of molecular catalytic sets exhibit dynamics of on-off changing states) and, on the other hand, for global self-regulations of the metabolic transitions (the dynamical reorganizations of the patterns during the changes provoked by the external perturbations).

We have used ApEn to estimate the degree of complexity and of information contained in the local attractors. Approximate Entropy is also a measure of the self-organization of a system and estimates the rate at which information about the state of a system is lost.

In this sense, the results have shown that in the numerical series the entropy is low indicating a high level of predictability and information in the local attractors which govern the corresponding transition patterns. The entropy values of the numerical metabolic series exhibit a narrow range of low values (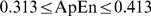).

In our opinion, the small deviation appears because in the model, in order to simplify, the flux integration functions and the regulatory signal integration functions of all metabolic subsystems are quite similar. In this sense, we think that the experimental metabolic data in cellular conditions should show notably different entropy values.

The emergence of local attractors belonging to different metabolic subsystems has been investigated in extensive studies mainly carried out by means of systems of differential equations, e.g., in Krebs cycle [96], amino acid biosynthetic pathways [97], oxidative phosphorylation subsystem [98], glycolytic subsystem [99], transduction in G-protein enzyme cascade [100], gene expression [101], cell cycle [102], etc.

From the biochemical point of view, these metabolic processes represent enzymatic sets self-organized as dissipative dynamical systems which carry out their activity relatively independently between them and play distinctive, systematic and essential roles in the cell [25], [29].

In previous works, we have also investigated the emergence of dynamical patterns depending on different input flux values in a glycolytic subsystem model which is governed by means of a system of differential equations with delay [103]. In these studies we have analyzed different attractor dynamics linked to Hopf bifurcations [104], tangent bifurcations [105] and the classical period-doubling cascade preceding chaos [106]. Likewise, we have also studied the multiplicity of coexisting attractors in the phase space [106].

In all these works, each metabolic subset forms a unique, absolutely well-defined, deterministic, dynamical system and the local variables are perfectly identified. In most of the cases, they have to do with the catalytic kinetics of the different enzymes belonging to each subsystem.

In our dissipative metabolic network model, each subsystem receives different number of fluxes and regulatory signals which, in all the cases, are output activities of other metabolic subsystems.

Therefore, each subsystem receives a subset of the total output activities developed by all the subsystems in the network in form of input fluxes and input regulatory signals.

The activity of each metabolic subsystem is determined by their local variables: on the one hand, the input flux and input regulatory signals and on the other hand the variables related to the catalytic kinetics of the different enzymes which have been substituted in our model by the variables belonging to the flux integration functions and regulatory signal integration functions.

However, the phase spaces of the global attractors are formed by the set of all subsystem output activities. Therefore, the variables of the flux integration functions and regulatory signal integration functions do not participate in these phase spaces.

Since both spaces of phases are formed by different variables, the global attractors and the local attractors are different and the local attractors cannot be considered as mere projections of a global attractor.

The results also show that, as a consequence of the emergent attractor dynamics, three essential properties can be observed: the **self-organization** of the global structure characterized by exhibiting a metabolic core, the **self-regulation** of the global structure which permits the integration of external stimuli adapting the system to them and the **persistent chaotic dynamics** for which the generated chaotic behaviours are correlated in time.

The global reactive structure emerges spontaneously in the dissipative metabolic network due to self-organized processes [68], [69]. As a consequence of dynamic and dissipative processes the metabolic system increases its complexity generating a new global structure that did not exist before and which is characterized by a set of metabolic subsystems which are always in an active state (metabolic core), while the rest of the enzymatic subsystems exhibit *on-off* changing states.

Self-organization is a spontaneous process, i.e., the complex system abandoned to itself is ordered in an immediate way, emerging without necessity of an external source of information and does not depend on local properties of subsystems.

The fundamental factor for the spontaneous emergence of the global self-organized enzymatic structure characterized by a metabolic core is the number of metabolic subsystems in the networks (see our paper [69]). This structure for a sufficient number of metabolic subsystems emerges independently of: the proportion of the allosteric and covalent enzymes present in the networks, the number of substrate fluxes and regulatory signals per catalytic element, the changes in the values of the outer fluxes, as well as the topology of all flux and regulatory signal interconnections [69].

Self-organization is a different property from self-regulation and it can be understood as a global process by which the metabolic system tends to reach particular activity states or a set of cycling activity states with autonomy from external factors.

Self-regulation permits the integration of external stimuli and the adaptation of the system to them. As a consequence of this process, the metabolic subsystems adopt different functional activities exhibiting distinct but inter-related and coordinated catalytic behaviours.

This process is also an emergent property originated by the complex dynamics of the global interactions in the metabolic system and implies the modulation of each metabolic subsystem activity, driving the whole catalytic behaviours over time and across changing circumstances.

In our opinion, it seems that the metabolic processes forms a structure as a whole, highly interconnected, able to transmit information between its parts, in such a way that the activity of every metabolic subsystem could be considered as an informative operation. Each catalytic element of the network, in its subordination to signals generated by other metabolic subsystems, would perform three functions at the same time: signal reception, signal integration and acting as a source of information.

The transmission of information between the metabolic subsystems forces them to be interlocked between themselves; i.e., each subsystem is conditioned to cooperate and have precise and specific activity regimes, regulating their local activities forming to the global activity of the metabolic network.

The dynamic net of regulatory interconnections (substrate flux, allosteric and covalent signals) acts like a “dynamic network of functional links” which defines in every moment a set of instructions that makes each subsystem evolve with a particular and precise catalytic pattern. As a consequence of these complex processes the metabolic structure acts as a whole being able to self-regulate.

Contrarily to self-organization, the self-regulation does not seem to be a spontaneous process since it depends on local properties of subsystems (fundamentally on the emergent attractor dynamics). Self-organization requires processing the information relative to the different states of all catalytic elements of the network.

Metabolic subsystems are enzymatic sets self-organized as dissipative dynamical systems which operate in far from equilibrium conditions forming a catalytic entity as a whole. They carry out their activities relatively independently between them and they play distinctive, systematic and essential roles in the cell.

The metabolic network is a dynamic complex super-structure which integrates different dynamic systems (the metabolic subsystems) and it forms a global and a unique, absolutely well defined, deterministic, dynamical system in which self-organization, self-regulation and persistent properties emerge.

Finally, we think that the existence of chaotic patterns long-term memory properties in the activity of the metabolic subsystems integrated in a stable global functional structure may constitute a biological advantage.

Chaotic patterns exhibit sensitive dependence on initial conditions. Sensitivity means that a small change in the initial state will lead to large changes in posterior system states and the fluctuations of the chaotic patterns are conditioned by the degree of perturbation on the initial conditions. These changes in the system states present exponential divergence, provoking fast separations in the chaotic orbits.

For “slow dynamical systems” the typical time scale of the chaotic fluctuations is on the order of 1
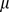
s [107], [108] and in “very fast chaotic systems” the characteristic time scale is on the order of 1 ns [107], [109].

On the other hand, different studies have shown that chaos permits fast transmission of information and high efficiency [110].

The existence of chaos in some metabolic processes may constitute a biological advantage by allowing fast and specific responses during the adaptation of the metabolic system to environmental perturbations.

For example, calcium plays an important role in the regulation of cell metabolism, modulating many physiological processes [111]. In response to extra cellular signals, the cytosolic calcium exhibits chaotic transitions which are conditioned by the intensity and type of the perturbation factor [112]. Since many enzymes are modulated by calcium, when intracellular calcium concentration presents chaotic patterns, they exhibit sensitivity to initial conditions and long-term memory properties which may influence the dynamical activities of the metabolic subsystems on those it acts, permitting fast and specific metabolic responses during the adaptation to external perturbations.

In this sense, numerous works have shown chaotic behaviours at cellular conditions (e.g., in intracellular free amino acid pools [31], respiratory metabolism [46], photosynthetic reactions [61], glycolysis [113], Krebs cycle [114], peroxidase-oxidase reactions [60], membrane potential [115], nuclear translocation of the transcription factor [58], NAD(P)H concentration [116], cyclic AMP concentration [117], ATP concentration [45], intracellular calcium concentration [112]).

Since a notable part of the biological temporary processes seem to be chaotic in cell conditions, it will be fundamental to take into account these persistent phenomena in quantitative biology.

The conception of a stable cellular metabolic structure able to self-organize spontaneously, forming a metabolic core of reactive processes that remain active under different growth conditions, able to self-regulate the transitions between the different molecular catalytic patterns and able to permit fast and specific responses during adaptation to the external medium may help to better understand cytological phenomena and to reinterpret them in a way closer to reality.

Understanding the elemental principles governing the global cellular metabolic structure as well as their nexus with central cytological processes may be one of the most important goals of the post-genomic era.

## Supporting Information

Table S1 Parameters of the dissipative metabolic network. MSb: the number of metabolic subsystems; Reg. Sign. Coef.: Coefficient values of the regulatory signals; Flux Parameter 1°: integration function parameters belonging to the first flux of the subsystems; Flux Parameter 2°: integration function parameters belonging to the second flux of the subsystems; Fluxes in: the topology of flux interconnections; Reg. Signals: the topology of regulatory signals (+, allosteric activation; −, allosteric inhibition; -T, covalent modulation of total inhibition).
(0.08 MB DOC)
