# Supplementary material for: Global Self-Regulation of the Cellular Metabolic Structure
Source: PLoS One. 2010 Mar 2;5(3):e9484. doi: 10.1371/journal.pone.0009484 (PMC2830472; doi:10.1371/journal.pone.0009484)
Supplement: Table S1 — Parameters of the dissipative metabolic network. MSb: the number of metabolic subsystems; Reg. Sign. Coef.: Coefficient values of the regulatory signals; Flux Parameter 1°: integration function parameters belonging to the first flux of the subsystems; Flux Parameter 2°: integration function parameters belonging to the second flux of the subsystems; Fluxes in: the topology of flux interconnections; Reg. Signals: the topology of regulatory signals (+, allosteric activation; −, allosteric inhibition; -T, covalent modulation of total inhibition). (0.08 MB DOC) [file pone.0009484.s001.doc]

Table S1

| MSb | Reg. Signals | | | | | | Reg. Sign. Coef. | | | | | | Reg. Sign. Coef. | | | | | | Reg. Sign.Coef. | | | | |
| --- | --- | --- | --- | --- | --- | --- | --- | --- | --- | --- | --- | --- | --- | --- | --- | --- | --- | --- | --- | --- | --- | --- | --- |
| 1 | 5 + | | 6 - | | 8 T | | .73 | | .29 | | .81 | | .37 | | .28 | | .60 | | .09 | | .47 | | .14 |
| 2 | 3 T | | 4 + | | 10 - | | .02 | | .68 | | .65 | | .44 | | .37 | | .58 | | .32 | | .06 | | .82 |
| 3 | 12 + | | 1 T | | 3 - | | .12 | | .46 | | .35 | | .98 | | .58 | | .64 | | .39 | | .03 | | .80 |
| 4 | 2 T | | 12 - | | 1 + | | .33 | | .22 | | .19 | | .11 | | .05 | | .56 | | .48 | | .90 | | .04 |
| 5 | 4 - | | 5 - | | 12 + | | .81 | | .70 | | .40 | | .31 | | .23 | | .68 | | .77 | | .29 | | .25 |
| 6 | 7 T | | 2 - | | 5 + | | .47 | | .52 | | .29 | | .07 | | .64 | | .71 | | .15 | | .92 | | .23 |
| 7 | 8 - | | 3 T | | 4 T | | .09 | | .19 | | .07 | | .74 | | .15 | | .08 | | .66 | | .18 | | .79 |
| 8 | 5 + | | 10 T | | 2 - | | .17 | | .93 | | .21 | | .45 | | .39 | | .87 | | .50 | | .65 | | .01 |
| 9 | 1 T | | 12 + | | 9 T | | .55 | | .11 | | .25 | | .40 | | .71 | | .16 | | .43 | | .46 | | .74 |
| 10 | 6 T | | 11 + | | 8 + | | .60 | | .16 | | .19 | | .86 | | .73 | | .65 | | .61 | | .02 | | .36 |
| 11 | 9 - | | 7 T | | 2 + | | .82 | | .50 | | .48 | | .63 | | .29 | | .01 | | .16 | | .24 | | .68 |
| 12 | 11 + | | 8 - | | 7 - | | .01 | | .18 | | .57 | | .56 | | .34 | | .21 | | .37 | | .41 | | .62 |
| MSb | | Fluses in | | | | Flux Parameter 1º | | | | | | Flux Parameter 2º | | | | | | Initial Conditions | | | | | |
| 1 | | 5 | | 7 | | .78 | | .86 | | .25 | | .83 | | .40 | | .61 | | .47 | | .09 | | .83 | |
| 2 | | 12 | | 1 | | .10 | | .75 | | .66 | | .49 | | .99 | | .98 | | .23 | | .23 | | .98 | |
| 3 | | 5 | | 10 | | .05 | | .57 | | .57 | | .62 | | .34 | | .55 | | .15 | | .56 | | .95 | |
| 4 | | 6 | | 2 | | .26 | | .91 | | .90 | | .87 | | .02 | | .18 | | .30 | | .74 | | .51 | |
| 5 | | 3 | | 11 | | .33 | | .84 | | .77 | | .17 | | .34 | | .96 | | .54 | | .60 | | .05 | |
| 6 | | 2 | | 4 | | .64 | | .83 | | .53 | | .25 | | .50 | | .73 | | .33 | | .92 | | .88 | |
| 7 | | 10 | | 3 | | .52 | | .58 | | .49 | | .08 | | .88 | | .61 | | .06 | | .47 | | .28 | |
| 8 | | 1 | | 7 | | .87 | | .05 | | .18 | | .51 | | .95 | | .43 | | .99 | | .71 | | .14 | |
| 9 | | 12 | | 12 | | .07 | | .02 | | .44 | | .37 | | .06 | | .36 | | .67 | | .72 | | .80 | |
| 10 | | 9 | | 8 | | .81 | | .40 | | .30 | | .91 | | .10 | | .58 | | .44 | | .03 | | .28 | |
| 11 | | 10 | | 9 | | .44 | | .98 | | .06 | | .71 | | .73 | | .94 | | .11 | | .66 | | .07 | |
| 12 | | 7 | | 1 | | .57 | | .32 | | .04 | | .18 | | .61 | | .22 | | .40 | | .27 | | .57 | |
